# Melatonin During Pre-Maturation and Its Effects on Bovine Oocyte Competence

**DOI:** 10.3390/antiox14080969

**Published:** 2025-08-07

**Authors:** Laryssa Ketelyn Lima Pimenta, Nayara Ribeiro Kussano, José Eduardo Vieira Chaves, Hallya Beatriz Sousa Amaral, Maurício Machaim Franco, José Felipe Warmling Sprícigo, Margot Alves Nunes Dode

**Affiliations:** 1 Institute of Biological Sciences, University of Brasilia, Brasilia 70910-900, Brazil; 2 School of Veterinary and Zootechnics, Federal University of Goiás, Goiânia 74690-900, Brazil; 3 Research Support Foundation of the Federal District, Brasilia 70635-815, Brazil; 4 Departament of Animal Science, University of Brasilia, Animal Science, Brasilia 70910-900, Brazil; 5 Departament of Veterinary Medicine, Federal University Uberlândia, Uberlândia 38400-902, Brazil; 6 Institute of Genetics and Biochemistry, Federal University Uberlândia, Uberlândia 38408-100, Brazil; 7 Animal Reproduction Laboratory, Embrapa Genetic Resources and Biotechnology, Brasilia 70770-900, Brazil

**Keywords:** antioxidant, biotechniques, biotechnology, meiosis, qPCR

## Abstract

To minimize the deleterious effects of oxidative stress and improve oocyte competence, we assessed the impact of melatonin during in vitro pre-maturation (pre-IVM) in bovine cumulus–oocyte complexes (COCs). We compared three groups: control (conventional IVM), pre-IVM control (without melatonin), and pre-IVM + MTn (with melatonin). The analyses included levels of reactive oxygen species (ROS), mitochondrial activity, oocyte lipid content, and the expression of genes related to oxidative stress and lipid metabolism in oocytes and cumulus cells. We also examined embryo quality by evaluating kinetics of development and gene expression. The pre-IVM + MTn group exhibited an increase (*p* ≤ 0.05) in ROS levels and a decrease (*p* ≤ 0.05) in lipid content, while maintaining mitochondrial activity similar (*p* > 0.05) to that of the control group. Regarding gene expression, the effect of pre-IVM, independent of melatonin, was characterized by a decrease in FABP3 transcripts in cumulus cells and reductions in GSS and NFE2L2 transcripts in oocytes (*p* ≤ 0.05). The pre-IVM + MTn group also displayed a decrease (*p* ≤ 0.05) in CAT and SOD2 transcript levels. In terms of embryonic development, the pre-IVM + MTn group achieved a higher blastocyst rate on D7 (*p* ≤ 0.05) compared to the control group (30.8% versus 25.8%), but with similar rates (*p* > 0.05) to the pre-IVM control group (30.8% versus 35.9%). However, there was a decrease in the levels of the PLAC8 transcript. This study indicates that, under the conditions tested, melatonin did not significantly benefit oocyte competence.

## 1. Introduction

Oocytes commonly used for in vitro embryo production (IVP) are typically obtained from antral follicles that have not yet completed their natural developmental process. As a result, these oocytes may be less competent when undergoing the complete maturation process [1]. This reduced competence can negatively impact IVP outcomes, as the efficiency of embryo development largely depends on the quality and developmental potential of the oocytes.

When oocytes are removed from the follicular environment, they spontaneously resume meiosis and progress to metaphase II, a stage indicative of nuclear maturation [2]. However, the cytoplasmic maturation process, which includes the redistribution of organelles and protein synthesis, may not occur effectively, potentially compromising the developmental competence of the oocytes [3,4]. Additionally, once retrieved, and placed in vitro conditions, oocytes undergo changes in redox potential, partly due to the loss of antioxidant defense from the follicular fluid and an increased production of reactive oxygen species (ROS). Factors such as exposure to light, oxygen concentrations, and medium composition, which differ significantly from the optimal in vivo environment, contribute to this oxidative imbalance. At the molecular level, ROS such as superoxide anion (O_2_^−^), hydrogen peroxide (H_2_O_2_), and hydroxyl radicals (•OH) are primarily generated in the mitochondria through electron leakage from the electron transport chain during oxidative phosphorylation, a process that becomes exacerbated in in vitro environments due to metabolic stress and reduced antioxidant defenses [5,6]. Another factor that can adversely impact oocyte quality is the lipid accumulation during the in vitro maturation (IVM) phase [7], which can elevate oxidative stress by promoting the lipoperoxidation of free fatty acids [8]. Together, these conditions can impair the oocyte’s ability to support subsequent embryonic development.

To overcome these limitations and enhance the quality of oocytes used for IVP, several strategies have been explored. One such approach involves the use of a pre-maturation methodology, which has been extensively studied in various species [9,10,11,12,13] and recently applied in the in vitro maturation of human oocytes [14,15,16,17]. This technique consists of a culture system—referred to as pre-in vitro maturation (pre-IVM or PIVM)—that precedes the conventional IVM phase. The goal is to provide oocytes with additional time after removal from the follicular environment, by blocking meiosis resumption and prolonging gap junction communication between oocytes and cumulus cells (CCs), thereby continuing the oocyte’s developmental programming [12,18]. Although pre-IVM systems in bovines have not consistently improved embryo development rates [18,19,20,21], few studies have explored the impact of offering optimized conditions during this meiotic arrest period.

In this context, another promising strategy is to optimize the oocyte’s environment immediately after retrieval. Mimicking in vivo conditions in the IVM medium, particularly through antioxidant supplementation, can help protect oocytes from oxidative stress induced by elevated ROS levels in the in vitro culture [6]. Among various antioxidants, melatonin has been extensively studied and shown to have protective effects during IVM across several species, improving embryo outcomes in goats [22,23,24], swine [25,26,27], rodents [28,29,30,31], sheep [32], and bovine [33,34].

Melatonin (N-acetyl-5-methoxytryptamine) is a hormone secreted by the pineal gland in mammals and acts as a broad-spectrum antioxidant [35,36]. During in vivo development, melatonin and other substances are secreted into the follicular fluid by granulosa cells, contributing to the nutrition and quality of oocytes, but these protections are lost during in vitro culture [37]. In bovine IVM systems, melatonin has been reported to reduce ROS levels, increase glutathione (GSH) and ATP content, promote proper organelles distribution, improve mitochondrial function, and regulate the expression of genes involved in antioxidant defense, cumulus expansion, and anti-apoptotic pathways [38,39,40,41,42,43,44,45,46]. In pigs, besides its cytoplasmic benefits, melatonin has also been shown to regulate lipid metabolism via receptor-mediated pathways, maintaining a balance between lipolysis and lipogenesis [47,48,49]. Although numerous studies have explored the role of melatonin during IVM, none have evaluated its effects during the pre-IVM phase in cattle. However, the benefits of melatonin use during pre-IVM have been reported for oocytes obtained from early antral follicles, after undergoing in vitro growth, also supplemented with melatonin [50].

Considering the individual benefits of both pre-IVM and melatonin supplementation, combining these two strategies may represent a promising approach to improve oocyte quality for IVP. Pre-IVM offers oocytes additional time to acquire developmental competence by temporarily maintaining meiotic arrest, while melatonin, with its antioxidant and regulatory properties, may protect and support cellular processes during this sensitive window. The presence of melatonin during pre-IVM could help preserve redox balance and promote a more synchronized progression of cytoplasmic and nuclear maturation, an essential factor for improving oocyte competence. Therefore, we hypothesize that melatonin supplementation during the pre-IVM phase can enhance oocyte quality and developmental potential and consequently improve the rate of bovine embryo development and embryo quality.

## 2. Materials and Methods

The reagents used were purchased from Sigma Aldrich (St. Louis, MO, USA) unless otherwise stated.

### 2.1. Experimental Design

To assess whether melatonin supplementation during pre-IVM could improve oocyte quality, several key parameters were evaluated. After retrieval and selection, COCs were randomly allocated to three treatment groups: (1) control: COCs matured in vitro for 24 h; (2) pre-IVM control: COCs underwent pre-IVM for 6 h, followed by 24 h of IVM; and (3) pre-IVM + MTn: COCs pre-matured with melatonin for 6 h, followed by 24 h of IVM.

Initially, we assessed the following morphological and functional parameters: nuclear maturation, ROS quantification, lipid content, mitochondrial activity, and gene expression. For this, COCs from all treatment groups were collected at specific time points: 0 h for the immature control (IC) and 24 h for the mature control (MC); after 6 h of pre-IVM and after 24 h of IVM for groups without melatonin supplementation (PMIC and PMMC) and with melatonin supplementation (PMMI and PMMM), respectively. At each time point, oocytes were denuded, fixed, stained, and analyzed using confocal or epifluorescence microscopy.

For gene-expression analysis, COCs were denuded, and oocytes and cumulus cells were then stored separately at −80 °C until further analysis. The quantification of genes associated with oxidative stress and lipid metabolism was performed in oocytes (*CAT*, *SOD1*, *SOD2*, *GSS*, *NFE2L2*, *PPARγ*, *CPT1A*, *ACSS2*, *FABP3*, and *PLIN2*) and in cumulus cells (*SOD1*, *SOD2*, *PPARγ*, and *FABP3*) by qPCR.

Subsequently, to evaluate the effect of treatment on oocyte competence, we monitored embryonic development. COCs underwent the four treatments described above and, post maturation, were co-incubated with sperm for 22 h. Zygotes were then cultured until day 7 (D7) of development. Cleavage rates were assessed on day 2 (D2, two days post fertilization), and blastocyst rates were evaluated on days 6 (D6) and 7 (D7). Embryos classified as expanded blastocysts on D7 were washed and stored in RNAlater at −80 °C for subsequent gene-expression analysis. The quantification of four genes associated with embryo quality (*PLAC8*, *KRT8*, *PRDX6*, and *SLC2A3*) was carried out by qPCR, using four pools of 15 embryos from the various treatments.

### 2.2. Recovery of Cumulus–Oocyte Complexes

Ovaries were obtained from a local slaughterhouse and transported to the laboratory at a temperature of 34 °C ± 2. They were then washed with a 0.9% sodium chloride solution supplemented with 100 μg/mL streptomycin sulfate and 100 IU/mL penicillin G. Follicles measuring between 3 and 8 mm were aspirated using 18 G needles attached to 10 mL syringes. COCs classified as grade I or II [51] were divided into three groups: control (conventional IVM), pre-IVM control [100 nM of natriuretic peptide precursor C, NPPC], and pre-IVM with the addition of melatonin [10^−9^ M]. The COCs from each group were washed and transferred to 150 μL drops of their respective medium, covered with mineral oil.

### 2.3. Pre-Maturation (Pre-IVM)

The pre-IVM medium was adapted from previously described methods [21]. It consisted of TCM-199 with Earle’s salts (Gibco^®^, Invitrogen, Carlsbad, CA, USA) supplemented with 0.075 mg/mL amikacin, 10% fetal bovine serum (FBS) (Gibco^®^), 0.68 mM L-glutamine, 1 mM sodium pyruvate, 0.1 μM cysteamine, and 10^−4^ IU/mL recombinant follicle-stimulating hormone (Gonal-F^®^, Merck Serono, Rockland, MA, USA). NPPC was added at a concentration of 100 nM with or without the addition of 10^−9^ M melatonin [52,53], diluted in DMSO. The COCs were incubated for 6 h at 38.5 °C in an atmosphere of 5% CO_2_, 5% O_2_, and 90% N_2_. After this period, the structures were transferred to the IVM medium.

### 2.4. In Vitro Maturation

The IVM medium consisted of TCM-199 with Earle’s salts (Gibco^®^) supplemented with 10% FBS (Gibco^®^), 0.01 IU/mL recombinant follicle-stimulating hormone (Gonal-F^®^, Merck Serono, Rockland, MA, USA), 0.1 mg/mL L-glutamine, 0.075 mg/mL amikacin, 0.1 μM cysteamine, and 0.2 mM sodium pyruvate [7]. IVM was conducted for 22–24 h at 38.5 °C under 5% CO_2_, 5% O_2_, and 90% N_2_.

### 2.5. In Vitro Fertilization and Culture

Post-IVM, COCs were washed and transferred to fertilization medium, Tyrode’s Albumin Lactate and Pyruvate (TALP), supplemented with 2 mM penicillamine, 1 mM hypotaurine, 250 μM epinephrine, and 10 μg/mL heparin. Frozen semen from a Nellore bull (*Bos taurus indicus*) was used for fertilization at a final concentration of 1 × 10^6^ sperm/mL. The gametes were co-incubated for 18–20 h. Subsequently, the presumptive zygotes were washed and transferred to synthetic oviduct fluid (SOF) culture medium supplemented with essential and non-essential amino acids, 0.35 mM sodium tricitrate, 2.8 mM myo-inositol [54], and 2.5% FBS (Gibco^®^). They were incubated under the same conditions previously mentioned. The cleavage rate was assessed on day 2 (D2) post-fertilization, and the blastocyst rate on days 6 (D6) and 7 (D7). Embryos classified as expanded blastocysts on D7 were washed with PBS and stored in RNAlater at −80 °C.

### 2.6. Gene Expression

Gene expression was analyzed using four pools of twenty oocytes, cumulus cells from twenty oocytes collected before and after IVM, and four pools of 15 expanded blastocysts collected on D7. Cumulus–oocyte complexes were denuded by successive pipetting in PBS without Ca^2+^ and Mg^2+^ for immature oocytes and in maintenance medium with 1% hyaluronidase for COCs post-IVM. After separating oocytes and cumulus cells, they were washed three times in PBS without Ca^2+^ and Mg^2+^ and subsequently stored in RNAlater at −80 °C until further use.

The extraction of total RNA from oocytes, cumulus cells, and blastocysts was performed separately using the RNeasy Plus Micro kit (Qiagen, Hilden, Germany) according to the manufacturer’s instructions. The extracted RNA was subsequently used for cDNA synthesis utilizing the GoScript Reverse Transcriptase Kit (Promega, Madison, WI, USA), also following the manufacturer’s guidelines. The first reaction involved an initial incubation at 70 °C for 5 min, followed by a step at 4 °C for 5 min. The second reaction consisted of an annealing step at 25 °C for 5 min, followed by an extension at 42 °C for 60 min and enzyme inactivation at 70 °C for 15 min.

The qPCR reactions were conducted using the GoTaq qPCR Master Mix Kit (Promega). For cumulus cells and blastocyst samples, the reaction conditions were set at 95 °C for 5 min, followed by 50 cycles of denaturation at 95 °C for 15 s, and annealing and extension at 62 °C for 30 s. For oocyte samples, the qPCR protocol was adjusted to 95 °C for 1 min, followed by 50 cycles of denaturation at 95 °C for 15 s, with annealing and extension at 60 °C for 1 min.

Each sample was analyzed in triplicate. The specificity of the PCR reactions was verified by examining the melting curves and the sizes of the amplicons on agarose gels. The nomenclature, primer sequences and concentrations, amplicon sizes, and GenBank accession numbers for each primer pair are detailed in Table 1. *GAPDH* was selected as the reference gene for normalizing the data.

### 2.7. Measurement of Reactive Oxygen Species Levels in Oocytes

Intracellular ROS levels in oocytes were quantified using H_2_DCFDA (Thermo Fisher, Waltham, MA, USA), dissolved in dimethyl sulfoxide (DMSO) and diluted in PBS with 1% BSA to a final concentration of 50 μM. Denuded oocytes were washed three times in PBS-BSA and then incubated in a 400 μL drop of H_2_DCFDA solution for 30 min at 38.5 °C in a dark environment. Subsequently, the oocytes were washed three times in PBS-BSA, transferred to a glass slide, and covered with mineral oil for fluorescence microscopy using a Zeiss^®^ Axiophot microscope (Carl Zeiss AG, Oberkochen, Germany). Fluorescence signal intensities (pixels) were quantified at a wavelength of 488 nm, with an excitation power of 9.14%, and emission recorded between 475 and 535 nm. Images were analyzed using ImageJ^®^ imaging software version 1.54 (National Institutes of Health, Bethesda, MD, USA).

### 2.8. Mitochondria, Lipids, and Chromatin Staining

Denuded oocytes were washed three times in PBS supplemented with 0.3% polyvinylpyrrolidone (PVP) as described previously and incubated for 30 min with MitoTracker Deep Red FM at 400 nM diluted in bench medium at a temperature of 35 to 37 °C. Afterward, the oocytes were washed three times in PBS-PVP and the methodology described previously for lipid staining was followed [7]. The oocytes were fixed for one hour in 4% paraformaldehyde, washed three times in PBS, and stored at 4 °C in 1% paraformaldehyde for up to seven days. Subsequently, they were washed twice in PBS-PVP and incubated for 30 min in PBS supplemented with 0.2% Triton X-100 at 25 to 27 °C. After three washes in PBS with 0.3% PVP, the oocytes were stained with Bodipy 493/503 (Molecular Probes, Eugene, OR, USA) at a concentration of 20 μg/mL (diluted in 50 μL of absolute ethanol and 950 μL of PBS) for one hour. The oocytes were then washed three times in PBS with 0.3% PVP, placed in 35 mm plates in 8 μL drops of anti-fade solution (SlowFade; Molecular Probes, Eugene, OR, USA), and examined under an LSM Leica Sp8 microscope (Leica microsystems, New Orleans, LA, USA). All samples were analyzed and photographed with a 20× objective and a 488 nm argon laser to visualize lipid droplets, with a fluorescence spectrum between 495 and 505 nm. For mitochondrial evaluation, a 638 nm laser was used, with excitation/emission of 644/665 nm. The oocytes underwent up to 20 transverse sections at 4 μm intervals. Z-stacking was employed to create an image of overlapping sections. After constructing the final image of each oocyte, they were adjusted to a grayscale (8-bit image) using the ImageJ program (National Institutes of Health), and the background was corrected. Mitochondrial activity was quantified by the mean number of pixels, and lipid measurements were expressed as a ratio of the area occupied by lipids (number of pixels) to the total area of the oocyte (μm).

After staining with MitoTracker and BODIPY, the oocytes were washed three times in PBS-PVP and then stained with Hoechst 33342 (10 mg/mL) for 15 min in the dark. Following staining, they were washed three times in PBS-PVP and mounted on slides in 8 μL drops of anti-fade solution (SlowFade; Molecular Probes). Chromatin conformation, indicating the meiotic stage, was assessed using fluorescence microscopy (Zeiss^®^ Axioplot). The chromatin was classified into stages: germinative vesicle (GV), germinative vesicle breakdown (GVBD), metaphase I (MI), and metaphase II (MII).

### 2.9. Statistical Analysis

The Shapiro–Wilk test was employed to assess the normality of the data. Parametric data were analyzed using either the ANOVA test or the unpaired *t*-test, while non-parametric data were evaluated using the Kruskal–Wallis or Mann–Whitney tests. The chi-squared test was applied to analyze the progression of meiosis and embryonic development. The relative expression of each gene was calculated using the ΔΔCt method with efficiency correction, as described previously [55]. Differences with a *p*-value of ≤0.05 were considered statistically significant. All analyses were conducted using GraphPad Prism software, version 8.0.1.

## 3. Results

### 3.1. Effect of Pre-Maturation on the Meiotic Progression of Oocytes

The meiotic progression of immature oocytes was assessed for the presence of GV or GVBD at 0 h and 6 h pre-IVM. No statistical difference (*p* > 0.05) was observed among the IC (20/20, 100%), PMIC (18/18, 100%), and PMMI (12/12, 100%) groups. Additionally, after IVM, the oocytes were evaluated for their ability to reach MII, and once again, no statistical difference (*p* > 0.05) was noted among the MC (17/18, 94.4%), PMMC (9/9, 100%), and PMMM (12/12, 100%) groups. Thus, pre-IVM treatment, whether in the absence or presence of melatonin, did not interfere with the oocytes’ ability to complete nuclear maturation.

### 3.2. Effect of Melatonin During Pre-Maturation on Reactive Oxygen Species Levels in Oocytes

**Figure 1:**
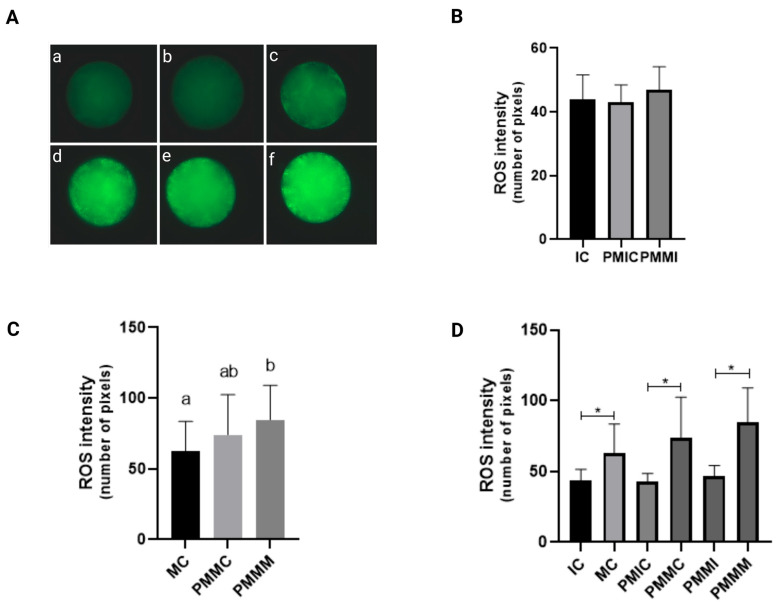
Representative images of bovine oocytes stained with H_2_DCFDA (**A**): Intracellular levels of reactive oxygen species in oocytes in different treatments at different time points. Immature control, 0 h ((**a**): IC; n = 25); pre-matured for 6 h ((**b**): PMIC; n = 26); pre-matured in the presence of melatonin for 6 h ((**c**): PMMI; n = 27); matured control, 24 h ((**d**): MC; n = 27); pre-matured for 6 h and matured for 24 h ((**e**): PMMC; n = 23); and pre-matured in the presence of melatonin for 6 h and matured for 24 h ((**f**): PMMM; n = 16). (**B**): immature groups; (**C**): matured groups; (**D**) before and after in vitro maturation. ^a,b^ Different superscripts or * between groups indicate significant differences (*p* ≤ 0.05).

The levels of ROS were measured in oocytes subjected to different treatments and collected at different time points (before and after IVM). Initially, the immature groups—the control (IC) and those retained for 6 h before maturation (PMIC and PMMI)—were compared. The results demonstrated that ROS levels were similar (*p* > 0.05) across all groups. However, upon evaluation after IVM, the group that underwent pre-IVM with melatonin (PMMI) exhibited higher ROS levels (*p* ≤ 0.05) compared to the MC group and similar levels to the PMMC group. When all groups were compared at 24 h of maturation, an increase in ROS levels (*p* ≤ 0.05) during IVM was observed across all groups (Figure 1).

### 3.3. Effect of Melatonin During Pre-Maturation on Mitochondrial Activity in Oocytes

**Figure 2:**
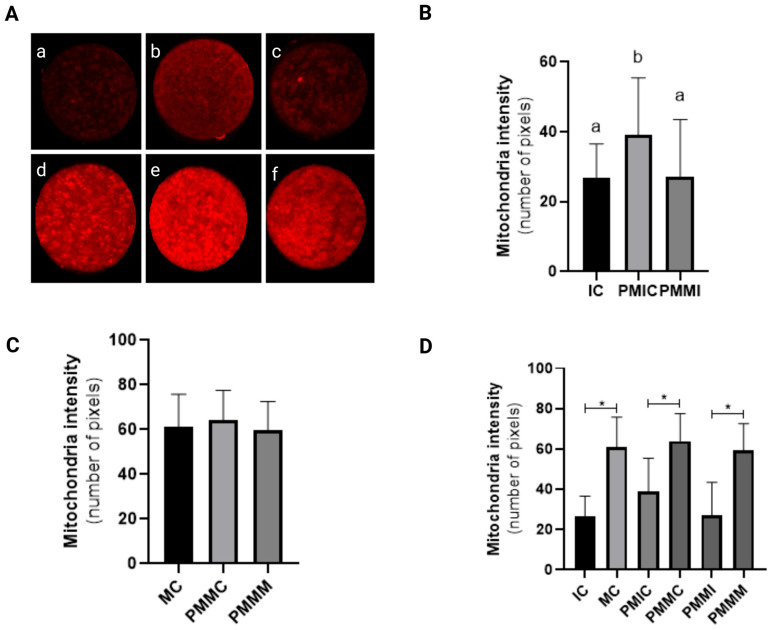
Representative images of oocytes stained with MitoTracker Deep Red (**A**). Level of mitochondrial fluorescence intensity in oocytes stained with MitoTracker Deep Red in the different groups. Immature control, 0 h ((**a**): IC; n = 32); pre-matured for 6 h ((**b**): PMIC; n = 31); pre-matured in the presence of melatonin for 6 h ((**c**): PMMI; n = 23); matured control, 24 h ((**d**): MC; n = 20); pre-matured for 6 h and matured for 24 h ((**e**): PMMC; n = 17); and pre-matured in the presence of melatonin for 6 h and matured for 24 h ((**f**): PMMM; n = 20); (**B**): immature groups; (**C**): matured groups; (**D**) before and after in vitro maturation. ^a,b^ Different superscripts or * between groups indicate significant differences (*p* ≤ 0.05).

Before IVM, oocytes from the PMIC group displayed higher mitochondrial fluorescence intensity (*p* ≤ 0.05) compared to those from the IC and PMMI groups. Following IVM, mitochondrial fluorescence levels were similar across all groups (MC, PMMC, and PMMM) (*p* > 0.05). Additionally, an increase in mitochondrial fluorescence intensity post-IVM was observed in all groups (*p* ≤ 0.05) when compared to their respective immature stages (Figure 2).

### 3.4. Effect of Melatonin During Pre-Maturation on the Lipid Content of Oocytes

**Figure 3:**
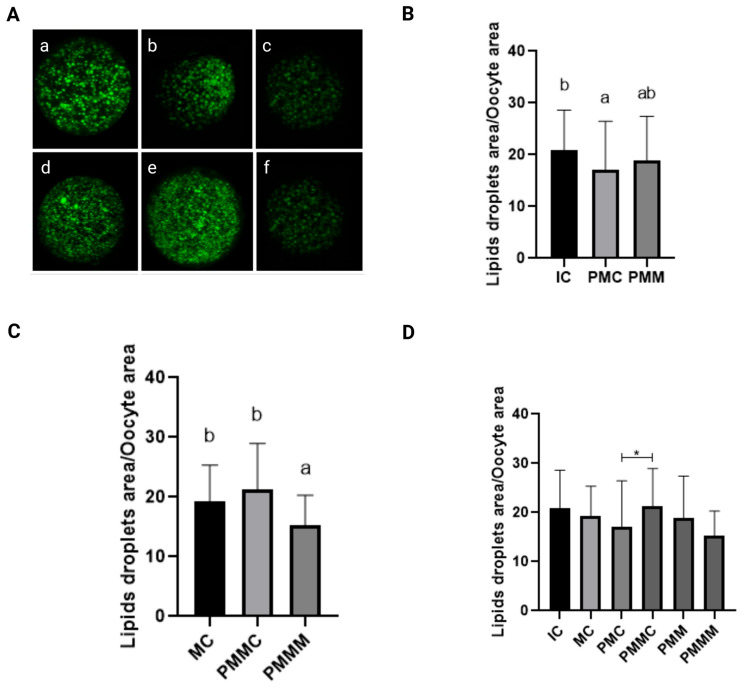
Representative images of oocytes stained with Bodipy 493/503 (**A**). Immature control, 0 h ((**a**): IC; n = 32); pre-matured for 6 h ((**b**): PMIC; n = 31); pre-matured in the presence of melatonin for 6 h ((**c**): PMMI; n = 23); matured control, 24 h ((**d**): MC; n = 20); pre-matured for 6 h and matured for 24 h ((**e**): PMMC; n = 17); and pre-matured in the presence of melatonin for 6 h and matured for 24 h ((**f**): PMMM; n = 20); Area of lipid droplets relative to the total area of the oocytes to evaluate the lipid droplets in the different groups. (**B**): immature groups; (**C**): matured groups; (**D**) before and after in vitro maturation. ^a,b^ Different superscripts or * between groups indicate significant differences (*p* ≤ 0.05).

Lipid content in oocytes was quantitatively assessed (Figure 3). Before IVM, oocytes from the pre-IVM control group (PMIC) exhibited a reduced quantity of lipid droplets (*p* ≤ 0.05) compared to the IC group. Their lipid levels were comparable (*p* > 0.05) to those in the group pre-matured with melatonin exposure. However, post 24 h of maturation, the group treated with melatonin displayed the lowest amount of lipid droplets (*p* ≤ 0.05).

### 3.5. Effect of Melatonin During Pre-Maturation on Gene Expression in Cumulus Cells and Oocytes

**Figure 4:**
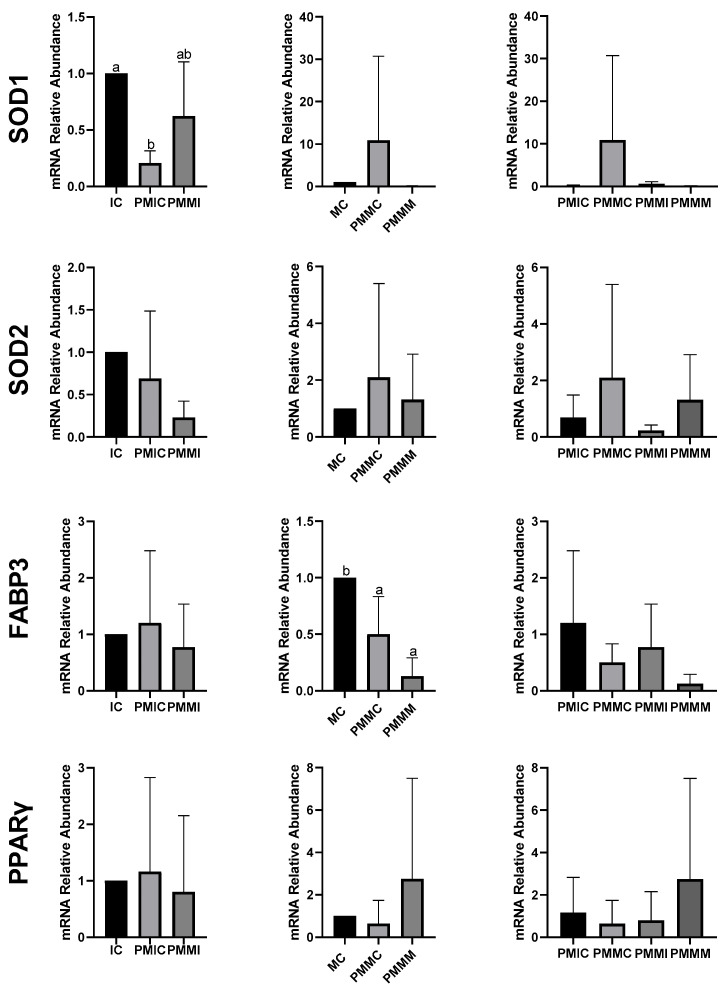
Relative mRNA levels of genes related to antioxidant defense (*SOD1* and *SOD2*) and lipid metabolism (*FABP3* and *PPARγ*) in cumulus cells of the cumulus–oocyte complex from different treatments and time points: immature control, 0 h (IC); pre-matured for 6 h (PMIC); pre-matured in the presence of melatonin for 6 h (PMMI); matured control, 24 h (MC); pre-matured for 6 h and matured for 24 h (PMMC); and pre-matured in the presence of melatonin for 6 h and matured for 24 h (PMMM). ^a,b^ Different superscripts indicate significant differences between groups (*p* ≤ 0.05).

The transcript levels of genes involved in antioxidant defense (*SOD1* and *SOD2*) and lipid metabolism (*FABP3* and *PPARγ*) were quantified in cumulus cells from all groups, before and after IVM. The results are depicted in Figure 4. The PMIC group showed lower transcript levels of the *SOD1* gene (*p* ≤ 0.05) compared to the IC group. However, transcript levels for the PMMI group were similar to those of the IC and PMIC groups. Post-IVM, similar (*p* > 0.05) transcript levels of *SOD1* were observed across the MC, PMMC, and PMMM groups. Regarding the *FABP3* gene, after maturation, transcript levels decreased in both groups subjected to pre-IVM, regardless of melatonin exposure (PMMC and PMMM), compared to the MC group (Figure 4).

**Figure 5:**
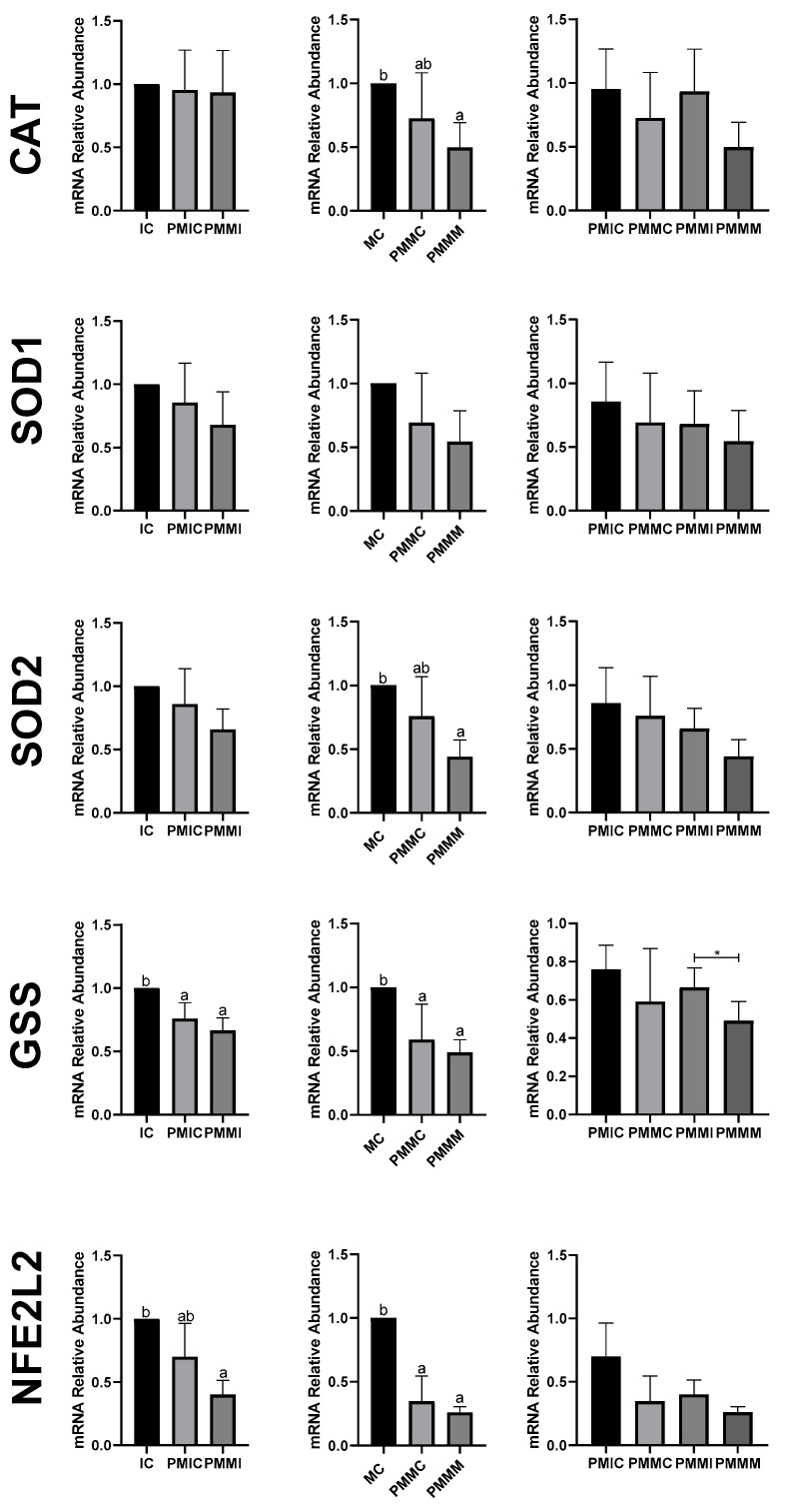
Relative mRNA levels of genes related to antioxidant defense (*CAT*, *SOD1*, *SOD2*, *GSS*, and *NFE2L2*) in oocytes in different treatments at different time points: immature control, 0 h (IC); pre-matured for 6 h (PMIC); pre-matured in the presence of melatonin for 6 h (PMMI); matured control, 24 h (MC); pre-matured for 6 h and matured for 24 h (PMMC); and pre-matured in the presence of melatonin for 6 h and matured for 24 h (PMMM). ^a,b^ Different superscripts or * between groups indicate significant differences (*p* ≤ 0.05).

In oocytes (Figure 5), transcript levels of the *GSS* gene were reduced (*p* ≤ 0.05) after 6 h of pre-IVM in both the PMIC and PMMI groups compared to the IC group. The level of *NFE2L2* transcripts decreased only in the PMMI group compared to the IC group. Post-IVM, transcript levels of *CAT* and *SOD2* were lower in the PMMM group compared to the MC group, while *GSS* and *NFE2L2* transcripts were reduced in both pre-IVM groups (PMMC and PMMM) relative to the control group (MC).

**Figure 6:**
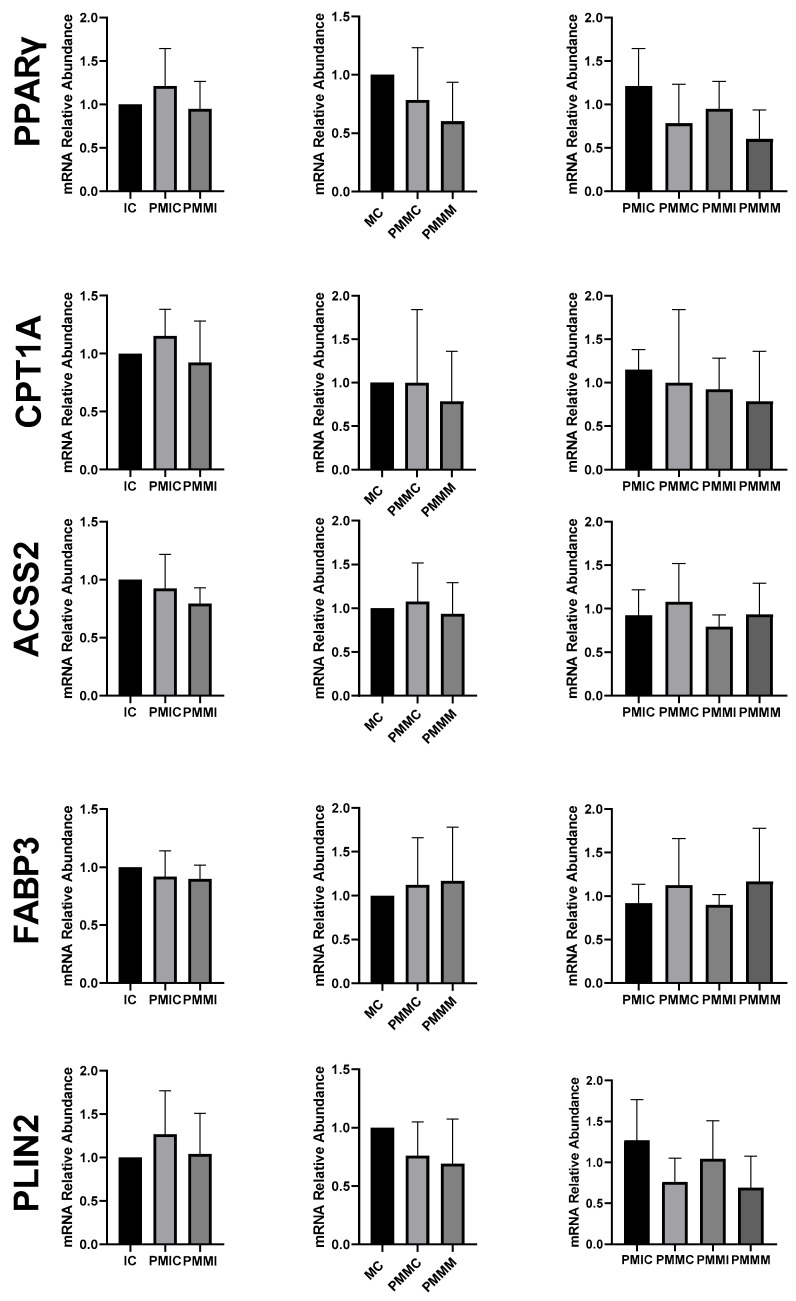
Relative mRNA levels of genes associated with lipid metabolism (*PPARγ*, *CPT1A*, *ACSS2*, *FABP3*, and *PLIN2*) in oocytes in different treatments at different time points: immature control, 0 h (IC); pre-matured for 6 h (PMIC); pre-matured in the presence of melatonin for 6 h (PMMI); matured control, 24 h (MC); pre-matured for 6 h and matured for 24 h (PMMC); and pre-matured in the presence of melatonin for 6 h and matured for 24 h (PMMM).

The expression of genes associated with lipid metabolism (*PPARγ*, *CPT1A*, *ACSS2*, *FABP3*, and *PLIN2*) did not differ (*p* > 0.05) among the oocytes of the evaluated groups (Figure 6).

### 3.6. Effect of Melatonin During Pre-Maturation on Embryonic Development

Embryonic development (Table 2) was evaluated by assessing 1752 COCs randomly assigned to different treatment groups. The COCs from the pre-IVM control and pre-IVM + MTn groups demonstrated superior (*p* ≤ 0.05) embryonic development compared to the control group in terms of cleavage rates at D2 (79.4% and 66.0%, respectively) and blastocyst formation at D7 (35.9% and 25.8%, respectively). However, at D6, the pre-IVM + MTn group exhibited a lower (*p* ≤ 0.05) blastocyst rate (14.1%) compared to the control (18.5%) and pre-IVM control (20.3%) groups. No differences were observed among groups in the different embryonic developmental stages at D6 or D7 (*p* > 0.05).

### 3.7. Effect of Melatonin During Pre-Maturation on Gene Expression in Blastocysts

**Figure 7:**
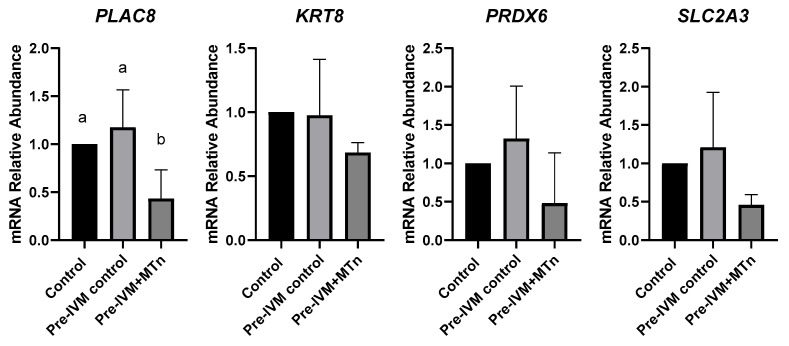
Relative mRNA levels of genes related to embryo quality (*PLAC8*, *KRT8*, *PRDX6*, and *SLC2A3*) in expanded blastocysts from three groups: control (in vitro maturation, 24 h); pre-IVM control (pre-IVM for 6 h and MIV for 24 h); and pre-IVM + MTn (pre-IVM in the presence of melatonin for 6 h, and MIV for 24 h). ^a,b^ Different superscripts indicate significant differences between groups (*p* ≤ 0.05).

To evaluate embryonic quality, the transcript levels of the *PLAC8*, *KRT8*, *PRDX6*, and *SLC2A3* genes were quantified in blastocysts from the three treatments (control, pre-IVM control, and pre-IVM + MTn). Only the *PLAC8* gene showed a significant difference between the investigated genes, with its transcription level reduced (*p* ≤ 0.05) in the pre-IVM + MTn group (Figure 7).

## 4. Discussion

Since oocytes spontaneously resume meiosis upon removal from the follicular environment, regardless of their developmental competence, many oocytes subjected to IVM are not fully competent to support embryonic development. To address this, a pre-IVM period can provide additional time for essential processes such as cytoplasmic reorganization and better synchronization with nuclear events. We hypothesized that enhancing the pre-IVM environment with melatonin, known for its potent antioxidant properties and potential regulation of lipid metabolism, could improve developmental competence. This hypothesis is supported by employing the physiological agent NPPC to delay meiosis for 6 h [21], alongside with melatonin at a concentration of 10^−9^ M established for bovine oocytes and embryos [46,52,53].

To investigate the effects of melatonin supplementation during meiotic arrest on oocyte quality, we initially evaluated intracellular ROS levels across experimental groups. Melatonin supplementation during the 6 h pre-IVM did not reduce ROS levels, as expected. Previous studies suggest that melatonin can improve oocyte quality and embryo development rate, mainly by reducing oxidative damage during IVM [41]. However, the beneficial effects of melatonin can be potentiated in conditions that cause high oxidative stress [35,44,56,57]. The use of a system with low oxygen tension, which reduces ROS production [58,59], may have constrained the additional antioxidant benefits of melatonin. Another contributing factor is that the 6 h period might not have been adequate to induce a reduction in ROS. In certain contexts, antioxidants like melatonin can have pro-oxidant effects, depending on the dosage and cellular environment, potentially increasing ROS levels instead of reducing them [60,61]. Moreover, an increase in ROS levels after IVM was observed in all groups. This increase can be attributed to various intrinsic and extrinsic factors related to the in vitro environment, which is more stressful than the physiological environment [62]. The exposure of oocytes to artificial conditions such as temperature, pH, medium composition, and atmospheric oxygen can induce ROS production [6].

The increase in ROS levels coincided with an increase in mitochondrial activity observed during IVM in all evaluated groups. During maturation, the oocyte’s energy demand increases, and the production of ATP through the mitochondrial electron transport chain results in the formation of ROS [63]. The efficiency of the repair mechanisms determines the amount of ROS [64]. The absence of significant differences between the groups after maturation indicates that melatonin did not interfere with mitochondrial activity.

Considering that melatonin, in addition to its antioxidant potential, is also associated with lipid metabolism [47,48], we evaluated whether its presence had any effect on oocytes. Melatonin supplementation did not affect lipid storage when immature oocytes were evaluated after the retention period. However, after IVM, oocytes exposed to melatonin exhibited the lowest concentration of lipids. This effect of melatonin was particularly evident when comparing the pre-IVM groups, which, despite having similar lipid content after retention, displayed a difference in content at 24 h of maturation, suggesting that these oocytes used lipids more efficiently to support the high energy metabolism necessary during maturation. Another possible explanation for the reduction in lipids could be that melatonin reduced the lipogenic capacity of the oocytes to store fatty acids in lipid droplets.

Lipids are essential for cytoplasmic maturation and provide the necessary energy during the early stages of embryonic development [65]. The storage of lipids in oocytes is a natural process, crucial for ensuring energy availability. However, the quantity and balance of these lipids are critical. Excess lipids in the oocyte cytoplasm can lead to problems that affect oocyte quality and embryonic development, such as oxidative stress, mitochondrial dysfunction, and abnormalities in cytoplasmic maturation [66]. In fact, oocytes matured in vitro have been shown to have a higher lipid content than those matured in vivo, indicating that the in vitro environment is more challenging and detrimental to oocyte quality [7].

To investigate the effect of melatonin, we analyzed the expression of genes involved in oxidative stress and lipid metabolism in cumulus cells and oocytes, both after retention and after IVM. Melatonin supplementation in the pre-IVM phase did not increase the transcript levels of genes associated with antioxidant defense (*CAT*, *SOD1*, *SOD2*, *GSS*, and *NFE2L2*) in oocytes at any of the time points evaluated. On the contrary, a reduction in *CAT* and *SOD2* levels was observed after IVM, as well as in GSS and NFE2L2 both before and after IVM, contrasting with studies that supplemented melatonin directly during IVM [43,46,52,67,68]. Although antioxidant transcript levels were already altered between the groups after pre-IVM, no phenotypic change was observed until the MIV phase, when energy demand increases, and modulation of gene expression adjusts ROS levels. These results suggest that, in the long term, transcript levels may play a determining role. However, this effect does not seem to be exclusive to melatonin supplementation, as the pre-IVM control group also showed similar results in terms of the abundance of mRNA transcripts and the level of ROS. It has been reported that C-type natriuretic peptide (CNP) treatment in pre-IVM increases mitochondrial activity and antioxidant defense by up-regulating genes [69], but this effect was not observed in the present study.

Regarding lipid metabolism, melatonin appears to have had little influence through gene regulation. The reduction in *FABP3* transcript levels in cumulus cells after IVM occurred in the pre-IVM groups, independently of the presence of melatonin. The *FABP* gene is responsible for encoding proteins that bind long-chain fatty acids and is associated with lipid accumulation during IVM, as well as with increased transcript levels in cumulus cells [70]. Although the group treated with melatonin at pre-IVM showed a slight reduction in lipid droplets, supplementation did not alter the expression of genes related to lipid metabolism (*PPARγ*, *CPT1A*, *FABP3*, *PLIN2*, and *ACSS2*). This result differs from those observed in porcine oocytes matured with melatonin, where the transcription of genes such as *PPARγ*, *PLIN2*, and *CPT1A* was increased [47,71].

Despite the apparent reduction in *FABP3* transcripts in cumulus cells, the lipid content of the oocytes in the pre-IVM control group remained similar to that of the post-IVM control group. This suggests that the decrease in lipid droplets observed in pre-matured oocytes treated with melatonin is not due to a lower transport of fatty acids mediated by *FABP3*, but rather to a reduced capacity to synthesize new droplets for energy storage. The increase in free fatty acids in the cytoplasm may contribute to the rise in ROS in oocytes pre-matured with melatonin due to lipoperoxidation, and not necessarily mitochondrial activity, as no statistical differences were present between the groups assessed by MitoTracker staining or in the levels of *CPT1A* gene transcripts.

The most critical assessment of the oocyte’s competence is its ability to cleave and develop into an embryo after IVF and IVC. Although the implementation of the pre-IVM phase generates controversial results regarding the improvement of embryonic development, sometimes showing no influence [18] or providing benefits [72,73], we observed that the pre-IVM groups had higher cleavage (D2) and blastocyst (D7) rates than the control group. This suggests that pre-IVM improved the quality of oocyte development and could be an attractive alternative to the low-oxygen tension system, given the inconsistent results on embryonic development in published works [74,75,76,77] and the low rates obtained in this study. Melatonin supplementation, on the other hand, did not enhance the beneficial effect observed with pre-IVM treatment.

Finally, embryonic quality was assessed by the expression of genes associated with placentation (*PLAC8* and *KRT8*), antioxidant defense (*PDRX6*), and cell metabolism (*SLC2A3*). Only the *PLAC8* gene showed a reduction in transcript levels. This gene is related to placentation, playing a role in placental development and maternal–fetal communication [78]. Its expression has been positively associated with D7 embryos that progress to a live birth [79] but contrasting results have been observed in high- and low-quality D14 embryos [80]. Thus, it is possible that melatonin during pre-IVM may have influenced later embryonic development.

## 5. Conclusions

We conclude that melatonin supplementation during pre-IVM had no beneficial effects on oocyte competence and bovine embryonic development. This result can be attributed to the brief exposure period of 6 h, considering that previous studies have shown positive effects of melatonin, such as the reduction in ROS and improvement in mitochondrial activity, when used throughout the 24 h IVM period in cattle. In addition, the role of melatonin as a regulator of lipid metabolism has been investigated predominantly in porcine oocytes, in IVM protocols with 41 h of exposure. This suggests that the duration and context of supplementation may be crucial factors in observing the potential benefits of melatonin. Although the mechanism by which pre-IVM affects gene regulation, especially in relation to antioxidant defense factors, is still unclear, pre-IVM treatment, independently of melatonin supplementation, showed promising results for embryonic development. This pre-IVM protocol could therefore be an interesting option for low-oxygen tension systems.

## Figures and Tables

**Table 1:** Information about the specific primers used for the amplification of gene fragments for the quantitative polymerase chain reaction analysis.

Gene	Full Name	Primer’s Sequence (5′-3′)	Cell Sample	Primer Concentration (nM)	Amplicon Size (bp)	GenBank Acession
*GSS*	Glutathione synthetase	F- GAGAGGGTGGAGGTAACAA	Oocyte	300	213	NM_001015630.1
		R- TCTTTCCCTCCCTGACATAG				
*NFE2L2*	NFE2 like bZIP transcription factor 2	F- GTCCAACCTTTGTCGTCATC	Oocyte	300	203	NM_001011678.2
		R- TCTACAGGGAATGGGATATGG				
*CPT1A*	Carnitine palmitoyltransferase 1A	F- GTTGCTGATGACGGCTATG	Oocyte	300	199	NM_001304989.2
		R- CCCAGAAGTGCTAAGAGATTTAC				
*CAT*	Catalase	F: GAA TGA GGA GCA GAG GAA AC	Oocyte	300	241	NM_001035386.2
		R: CTC CGA CCC TCA GAG ATT AG				
*PLIN2*	Perilipin 2	F- CGG CTA CGA TGA TAC AGA TG	Oocyte	300	200	NM_173980.2
		R- TGC GAA ACA CAG AGT AGA TG				
*ACSS2*	Acyl-CoA synthetase short chain family member 2	F: TGC ACC TGG ATT GCC TAA AAC	Oocyte	200	158	NM_001105339.1
		R: TTC ATT GGA TGG TCA AGC AGC				
*SOD1*	superoxide dismutase 1	F- GGGAGATACAGTCGTGGTAA	Oocyte and CCs	300	171	NM_174615.2
		R- CCAACATGCCTCTCTTCATC				
*SOD2*	Superoxide dismutase 2	F- GTG ATC AAC TGG GAG AAT GT	Oocyte and CCs	300	135	NM_ 201527
		R- AAG CCA CAC TCA GAA ACA CT				
*PPARy*	Peroxisome proliferator activated receptor gamma	F- GTCAGTACTGTCGGTTTCAG	Oocyte and CCs	300	200	NM_181024.2
		R- CAGCGGGAAGGACTTTATG				
*FABP3*	Fatty acid-binding protein 3	F: ATC GTG ACG CTG GAT GGC GG	Oocyte and CCs	200	210	NM_174313.2
		R: GCC GAG TCC AGG AGT AGC CCA				
*PLAC8*	Placenta associated 8	F: GAC TGG CAG ACT GGC ATC TT	Blastocyst	300	140	NM_016619
		R: CTC ATG GCG ACA CTT GAT CC				
*SCL2A3*	Solute carrier family 2 member 3	F: ACT CTT CAC CTG ATT GGC CTT GGA	Blastocyst	300	145	NM_174603.3
		R: GGC CAA TTT CAA AGA AGG CCA CGA				
*KRT8*	keratin 8	F: GGT TCT GGA GAC CAA ATG GAA	Blastocyst	300	97	NM_001033610.1
		R: CCG ACG GAG GTT GTT AAT GTA G				
*PDRX6*	Peroxiredoxin 3	F: GGC AGG AAC TTT GAT GAG AT	Blastocyst	300	205	NM_174643.1
		R: GTG TGT AGC GGA GGT ATT TC				
*GAPDH*	Glyceraldehyde-3-phosphate dehydrogenase	F: GGC GTG AAC CAC GAG AAG TAT AA	All cell types	300	118	NM_001034034.2
		R: CCC TCC ACG ATG CCA AAG T				

F = forward. R = reverse. CCs = cumulus cells.

**Table 2:** Embryonic development of pre-matured cumulus–oocyte complex in the presence of melatonin.

Group			Blastocyst (D6)				Blastocyst (D7)				
	Oocytes	Cleavage (D2)	eB	Bl	Bx	Total	eB	Bl	Bx	hB	Total
	n	n (%)	n (%)	n (%)	n (%)	n (%)	n (%)	n (%)	n (%)	n (%)	n (%)
IVM Control	594	392 (66.0) ^b^	54 (49.1)	45 (40.9)	11 (10.0)	110 (18.5) ^a^	14 (9.1)	44 (28.8)	92 (60.1)	3 (1.9)	153 (25.8) ^b^
pre-IVM Control	557	442 (79.4) ^a^	61 (54.0)	47 (41.6)	5 (4.4)	113 (20.3) ^a^	24 (12.0)	58 (29.0)	106 (53.0)	12 (6.0)	200 (35.9) ^a^
pre-IVM + MTn	601	451 (75.0) ^a^	51 (60.0)	29 (34.1)	5 (5.9)	85 (14.1) ^b^	19 (10.3)	57 (30.8)	99 (53.5)	10 (5.4)	185 (30.8) ^a^

^a,b^ Different letters in the same column indicate statistical differences (*p* ≤ 0.05), and no letters in the same column denote similar values (*p* > 0.05) between treatments. Pre-IVM control [100 nM of NPPC]. Pre-IVM + MTn [100 nM of NPPC and 10^−9^ M melatonin]. n: absolute total number; D2: two days post-fertilization; eB: early blastocyst; Bl: blastocyst; Bx: expanded blastocyst; hB: hatched blastocyst.

## Data Availability

The original contributions presented in this study are included in the article Further inquiries can be directed to the corresponding author.

## Abbreviations

The following abbreviations are used in this manuscript:

Pre-IVM	pre-in vitro maturation
COCs	cumulus-oocyte complexes
IVP	in vitro embryo production
ROS	reactive oxygen species
IVM	in vitro maturation
CCs	cumulus cells
Pre-IVM Control	COCs underwent pre-IVM for 6 h, followed by 24 h of IVM
Pre-IVM + MTn	COCs pre-matured with melatonin for 6 h, followed by 24 h of IVM
IC	immature control
MC	mature control
PMIC	COCs after 6 h of pre-IVM for groups without melatonin supplementation
PMMC	after 6 h of pre-IVM and after 24 h of IVM for groups without melatonin supplementation
PMMI	after 6 h of pre-IVM for groups with melatonin supplementation
PMMM	after 6 h of pre-IVM and after 24 h of IVM for groups with melatonin supplementation
D	days post-fertilization
NPPC	Natriuretic peptide precursor C
CNP	C-type natriuretic peptide
